# Blood biomarkers of Hikikomori, a severe social withdrawal syndrome

**DOI:** 10.1038/s41598-018-21260-w

**Published:** 2018-02-13

**Authors:** Kohei Hayakawa, Takahiro A. Kato, Motoki Watabe, Alan R. Teo, Hideki Horikawa, Nobuki Kuwano, Norihiro Shimokawa, Mina Sato-Kasai, Hiroaki Kubo, Masahiro Ohgidani, Noriaki Sagata, Hiroyuki Toda, Masaru Tateno, Naotaka Shinfuku, Junji Kishimoto, Shigenobu Kanba

**Affiliations:** 1 Department of Neuropsychiatry, Graduate School of Medical Sciences, Kyushu University, Fukuoka, Japan; 2 School of Business, Monash University, Jalan Lagoon Selatan, Bandar Sunway, Selangor Darul Ehsan, Malaysia; 3 VA Portland Health Care System, Portland, Oregon United States of America; 4 Department of Psychiatry, Oregon Health & Science University, Portland, Oregon United States of America; 5 Department of Psychiatry, National Defense Medical College, Tokorozawa, Saitama Japan; 6 Department of Neuropsychiatry, Sapporo Medical University, School of Medicine, Sapporo, Hokkaido, Japan; 7 International Center for Medical Research, Kobe University, Kobe, Japan; 8 Department of Research and Development of Next Generation Medicine, Faculty of Medical Sciences, Kyushu University, Fukuoka, Japan

## Abstract

*Hikikomori*, a severe form of social withdrawal syndrome, is a growing social issue in Japan and internationally. The pathophysiology of hikikomori has not yet been elucidated and an effective treatment remains to be established. Recently, we revealed that avoidant personality disorder is the most common comorbidity of hikikomori. Thus, we have postulated that avoidant personality is the personality underpinning hikikomori. First, we herein show relationships between avoidant personality traits, blood biomarkers, hikikomori-related psychological features, and behavioural characteristics assessed by a trust game in non-hikikomori volunteers. Avoidant personality traits were negatively associated with high-density lipoprotein cholesterol (HDL-C) and uric acid (UA) in men, and positively associated with fibrin degeneration products (FDP) and high sensitivity C-reactive protein (hsCRP) in women. Next, we recruited actual individuals with hikikomori, and compared avoidant personality traits, blood biomarkers, and psychological features between individuals with hikikomori and age-matched healthy controls. Individuals with hikikomori had higher avoidant personality scores in both sexes, and showed lower serum UA levels in men and lower HDL-C levels in women compared with healthy controls. This is the first report showing possible blood biomarkers for hikikomori, and opens the door to clarify the underlying biological pathophysiology of hikikomori.

## Introduction

“Hikikomori”, a severe form of social withdrawal seen more often in individuals of relatively young age, has become a growing social issue since around 1990 in Japan^1–4^. Hikikomori is usually defined as follows: (i) spending most of the day and nearly every day at home, (ii) avoiding social situations such as attending school or workplace, (iii) avoiding social relationships such as friendships or contact with family members, and (iv) significant distress or impairment due to social isolation (i to iii require duration of at least 6 months)^5^. An epidemiological study of hikikomori showed a lifetime prevalence of more than 1% in adults in Japan^2^. Although hikikomori had been previously thought to be proper only to Japanese society/culture^6^, recent international studies have revealed that hikikomori is found in various races and areas around the world including Hong Kong, urban areas of China, India, South Korea, Spain, and the United States^5–10^. Our recent international survey has shown that the most common comorbidity of hikikomori is avoidant personality disorder^11^. Hikikomori and avoidant personality disorder seem to have many psychological and behavioural features in common: shyness; ambivalent attachment styles and life experiences including rejection by peers and parents^12^; high loneliness and impaired social networks; apparent inability to maintain meaningful social ties^5^; social withdrawal and avoidance of real-world human interactions; and tendency toward indirect interpersonal exchanges via the Internet^13^. Urgent development of effective prevention and treatment methods for hikikomori is required. However, there has been neither preventive nor treatment measures of hikikomori, because the pathophysiology of hikikomori has not been fully elucidated yet.

Herein, we preliminary explored the underlying psychological and biological pathophysiology of hikikomori. First, we hypothesized that avoidant personality traits would be associated with hikikomori, and investigated the association between avoidant personality traits, blood biomarkers, behavioural characteristics, and psychological aspects in student-age non-clinical samples (Study 1). Interpersonal relationships in the real world are at least partially based on behaviours derived from unconscious decision-making^14–17^. To assess behavioural characteristics, we applied self-rated questionnaires and also a personal computer (PC)-based trust game, which can accurately evaluate unconscious decision-making and consequently estimate interpersonal relationships which participants have in the real world^16^. Second, we compared avoidant personality traits and candidate blood biomarkers for avoidant personality traits identified in Study 1 between actual individuals with hikikomori and age-matched healthy control subjects (Study 2).

In Study 1, we recruited a total of 101 young non-clinical volunteers in a university campus and collected their socio-demographic information. Forty-six participants were male, and no participant was excluded from this study. Participants’ demographic details are shown in Table 1. Each participant completed a series of self-rated questionnaires, played a PC based trust game, and provided a non-fasting venous blood sample.

**Table 1:** Demographic data.

**Study 1**.			
	male	female	*p* value
n	46	55	
age, median (IQR)	21.0 (4.0)	21.0 (2.0)	0.43^b^
nationality			
Japanese	44	55	0.12^a^
not Japanese (Asians)	2	0	
cohabitation			
living alone	33	35	0.39^a^
not living alone	13	20	
status			
current student	44	55	0.37^a^
worker	2	0	
**Study 2**.			
	**hikikomori**	**healthy control**	***p*** **value**
n, male/female	29/26	34/44	
age, median (IQR)			
male	38.0 (19.0)	35.0 (8.0)	0.61^b^
female	34.0 (12.0)	39.0 (17.0)	0.092^b^

^a^Chi-squared test, ^b^Welch’s t test or Mann-Whitney U test.

**Figure 1:**
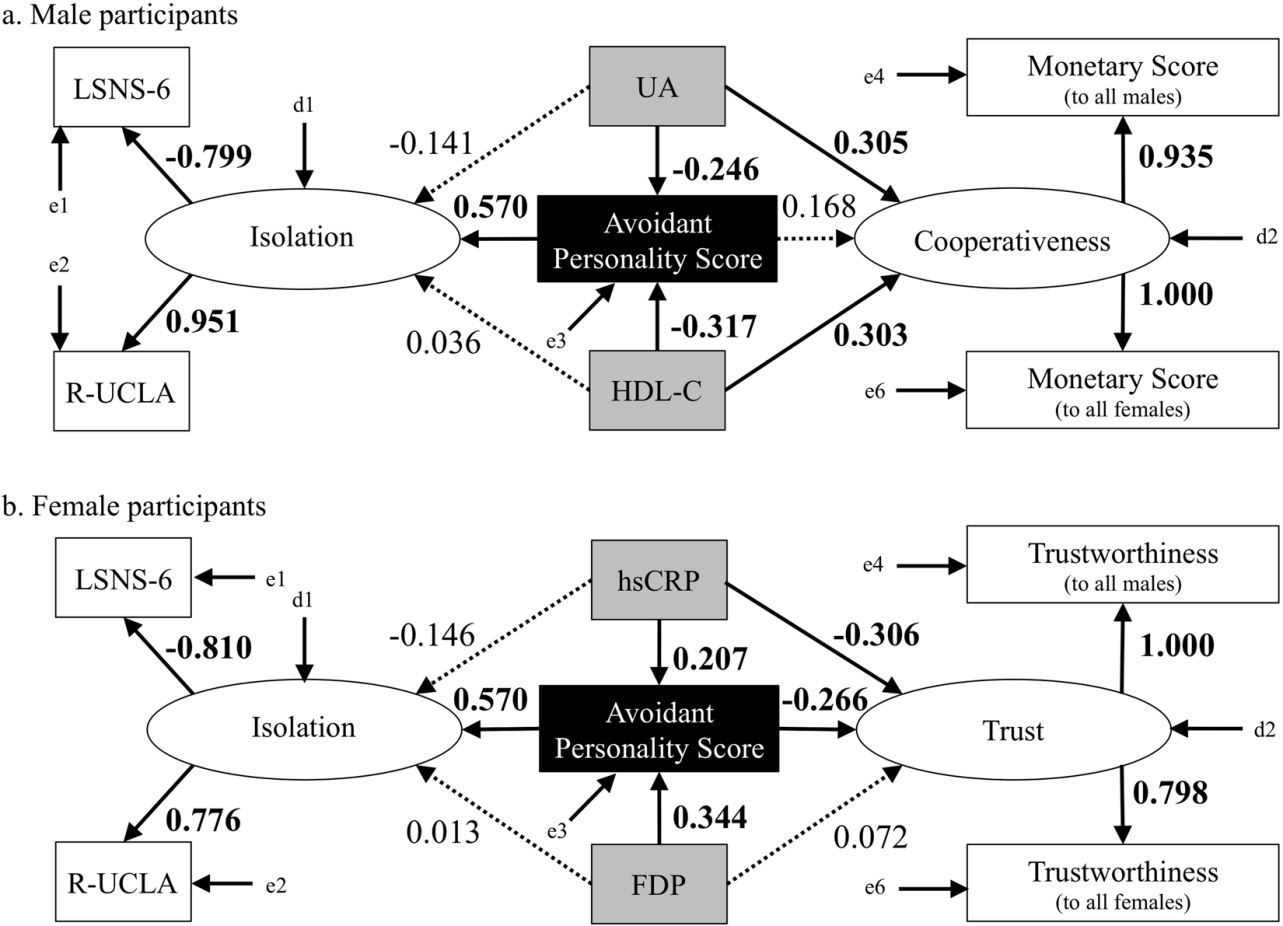
Structure equation models showing the connections between avoidant personality score, blood biomarkers, psychological features, and behavioural characteristics in non-hikikomori volunteers. The arrows with bold lines have a statistically significance or a trend, and broken lines have neither a statistically significance nor a trend. The numbers above the arrows show standardized path coefficients. e: error terms, d: disturbance terms.

First, we assessed causal relationships between avoidant personality score, blood biomarkers, behavioural characteristics, and psychometrics using structural equation modeling (SEM). Next, we examined the association between avoidant personality score and blood biomarkers using multiple regression analyses with stepwise method for efficient biomarkers detection. Finally, correlation analyses were performed between avoidant personality score, blood biomarkers, behavioural characteristics, and psychometrics. The best-fit models by gender are shown in Fig. 1. Good fits of each model were obtained as follows: χ2 = 8.143 (*p* = 0.62; statistically insignificance means a good fit), goodness of fit index (GFI) = 0.954, comparative fit index (CFI) = 1.000, Tucker–Lewis index (TLI) = 1.025, and the root mean square error of approximation (RMSEA) <0.001 in men; χ2 = 6.569 (*p* = 0.77), GFI = 0.968, CFI = 1.000, TLI = 1.070, RMSEA <0.001 in women. The SEM suggested that higher avoidant personality scores led to stronger isolation that increased loneliness and decreased social support and networks (i.e., high scores of the Revised University of California, Los Angeles Loneliness Scale (R-UCLA) and the Lubben Social Network Scale-6 (LSNS-6), respectively) in both genders. In men, lower serum high-density lipoprotein cholesterol (HDL-C) and serum uric acid (UA) were proposed to lead to higher avoidant personality scores (standardized path coefficient (β) = −0.317, *p* = 0.020; and β = −0.246, *p* = 0.072, respectively), and less cooperativeness (β = 0.303, *p* = 0.038; and β = 0.305, *p* = 0.033, respectively). However, the direct causality between avoidant personality score and cooperativeness was not shown (β = 0.168, *p* = 0.26). In women, higher plasma fibrin degeneration products (FDP) and serum high sensitivity C-reactive protein (hsCRP) are suggested to lead to higher avoidant personality score (β = 0.344, *p* = 0.006; and β = 0.207, *p* = 0.097, respectively). Furthermore, higher avoidant personality score and hsCRP tended to make female participants estimate others to be untrustworthy (β = −0.266, *p* = 0.051; and β = −0.306, *p* = 0.018, respectively).

Next, we examined the association between avoidant personality score and blood biomarkers using multiple regression analyses with stepwise method for efficient biomarkers detection. Regression analyses with stepwise method revealed that avoidant personality scores in men and women were predicted by HDL-C (β = −0.323, *p* = 0.028, Condition Index = 10.383) and FDP (β = 0.329, *p* = 0.014, Condition Index = 12.252), respectively.

Finally, correlation analyses were performed between avoidant personality score, blood biomarkers, behavioural characteristics, and psychometrics. Correlation analyses showed negative correlations between avoidant personality score and HDL-C, total bilirubin, and UA in men; and positive correlations between avoidant personality score and FDP and hsCRP in women (Supplementary Table 1). Although correlation analyses showed no significant associations between avoidant personality score and results of the trust game in men, significant negative correlations were found between avoidant personality score and extensive results of the trust game in women (Supplementary Table 2), suggesting that higher avoidant personality traits appeared to make female participants more mistrustful of and less attracted to others. Moreover, trust games showed that men with higher HDL-C or UA levels tend to be more cooperative (i.e., give more money to their partners). Women with higher hsCRP levels showed weaker trust in their partners, especially in male partners (Supplementary Tables 3–4). In both genders, participants with higher avoidant personality score were more likely to be lonely (i.e., higher scores of the Preference for Solitude Scale (PSS) and R-UCLA), socially anxious (i.e., higher score of the Mini Social Phobia Inventory (MINI-SPIN)), lacking social support (i.e., lower scores of LSNS-6 and the Multidimensional Scale of Perceived Social Support (MSPSS)), and mistrustful of others (i.e., lower score of the Yamagishi and Yamagishi’s trust scale (YYS)). Interestingly, avoidant personality score had a positive correlation with the Internet Addiction Test (IAT) only in male participants (Supplementary Table 1). In men, significant positive correlations were found between FDP and YYS. Negative correlations were shown between total bilirubin and PSS; and between UA and MINI-SPIN. In women, significant positive correlations were found between serum low-density lipoprotein cholesterol (LDL-C) and LSNS-6. A negative correlation was shown between total bilirubin and YYS or MSPSS (Supplementary Tables 3–4).

In accord with the results of Study 1, we recruited 55 individuals with hikikomori and 78 age-matched non-clinical volunteers as a healthy control group in Study 2. Twenty-nine individuals and 34 volunteers were male; and one individual with hikikomori was excluded from this study because of a history of traumatic brain injury. Participants’ demographic details are shown in Table 1. Each participant completed a series of self-rated questionnaires that were the same as in Study 1, underwent a structured interview for hikikomori with an experienced psychiatrist or a psychologist, and provided a non-fasting venous blood sample. The blood biomarkers measured were the same as in Study 1. First, we assessed normality of data, and then compared avoidant personality score and blood biomarkers between the two groups. Next, we assessed classifying efficiencies and potentials of blood biomarkers using canonical discriminant analysis and receiver-operating characteristic (ROC) curves. Finally, correlation analyses were performed between avoidant personality score, blood biomarkers, and psychometrics.

Avoidant personality score in individuals with hikikomori was significantly higher than in healthy controls in both genders (*p* < 0.001). Male individuals with hikikomori had significantly lower UA (*p* = 0.001), and female individuals with hikikomori had significantly lower HDL-C (*p* = 0.011) (Tables 2–3). We performed discriminant analysis using the diagnosis of hikikomori as the dependent variable, and UA in male participants and HDL-C in female participants as predictor variables based on the results of comparison of blood biomarkers between individuals with hikikomori and healthy controls. Moreover, to evaluate the diagnostic potential of the biomarkers, we constructed a ROC curve and then calculated the area under the curve (AUC) and 95% confidence interval (CI). The percentages of correct classification between hikikomori and healthy control groups were 72.4% with a sensitivity of 66.7% and a specificity of 76.5% in male participants, and 61.3% with a sensitivity of 61.1% and a specificity of 61.4% in female participants. AUC (95% CI) was 0.772 (0.647–0.897) with a sensitivity of 70.6% and a specificity of 75.0% (*p* < 0.001) in male participants, and 0.693 (0.550–0.835) with a sensitivity of 65.9% and a specificity of 61.1% (*p* = 0.018) in female participants (Supplementary Table 5). Although not significant, correlation analyses showed that UA was positively correlated with PSS (r = 0.379, *p* = 0.068) and MINI-SPIN (ρ = 0.404, *p* = 0.050) in male individuals with hikikomori. HDL-C was not correlated with avoidant personality score and any psychometrics in female individuals with hikikomori (Supplementary Tables 6–7).

**Table 2:** Comparison of avoidant personality score and blood biomarkers between male individuals with hikikomori and healthy control.

	hikikomori			healthy control			*p* value
	n	median/mean	IQR/SD	n	median/mean	IQR/SD	
avoidant personality score biomarkers	28	5.0	2.0	34	2.0	2.0	<0.001^a^
HDL-C	24	54.1	11.8	34	58.7	14.6	0.21^a^
LDL-C	24	106.0	48.0	34	118.5	38.0	0.64^a^
FDP	24	0	0	34	0	0	0.36^a^
total bilirubin	24	0.5	0.3	34	0.6	0.2	0.20^a^
UA	24	5.0	1.3	34	6.2	1.3	0.001^b^
hsCRP	24	342.0	620.0	34	387.5	828.0	0.99^a^

^a^Mann-Whitney U test, ^b^Student’s or Welch’s t test.

**Table 3:** Comparison of avoidant personality score and blood biomarkers between female individuals with hikikomori and healthy control.

	hikikomori			healthy control			*p* value
	n	median/mean	IQR/SD	n	median/mean	IQR/SD	
avoidant personality score	24	5.0	3.0	44	1.5	3.0	<0.001^a^
biomarkers							
HDL-C	18	64.7	11.1	44	73.3	12.2	0.011^b^
LDL-C	18	123.8	33.7	44	114.0	31.0	0.27^b^
FDP	14	0	0	43	0	0	0.14^a^
total bilirubin	18	0.50	0.25	44	0.55	0.30	0.42^a^
UA	18	4.50	1.70	44	4.00	1.45	0.53^a^
hsCRP	18	215.5	2137.0	44	213.5	572.0	0.14^a^

^a^Mann-Whitney U test, ^b^Student’s or Welch’s t test.

In sum, we herein investigated the biological pathophysiology of hikikomori by focusing on avoidant personality traits. Study 1 showed significant relationships between avoidant personality traits, blood biomarkers, and behavioural characteristics assessed by the trust game, and psychological features (loneliness, social anxiety, trust, and Internet addiction) in healthy volunteers. Candidate blood biomarkers of avoidant personality traits were identified as follows: HDL-C and UA in men, and FDP and hsCRP in women. The SEM analysis suggests that avoidant personality traits exacerbating psychological isolation in both sexes were indirectly associated with behavioural uncooperativeness in men, and directly induced distrust of others in women. Study 2 revealed that individuals with hikikomori had higher avoidant personality scores in both sexes, lower serum UA levels in men, and lower serum HDL-C levels in women.

Social relationships cannot be assessed only by self-rated questionnaires and interviews as social relationships in the real world are based on unconscious behavioural and emotional patterns, and usually have deviations from self-rated sociability itself. Therein, experimental economic games, developed in the fields of social psychology and economics, help offer insight into real-world behaviour and personality^16^. Economic games have been proposed as a novel candidate for an assessment tool of real-world interpersonal problems in patients with psychiatric disorders including personality disorders those who tend to have difficulties in appropriate decision-making^18^. Trust game is such an economic game and has been used to evaluate a person’s trust toward others^19^. Trust is a basis of cooperation in social interactions, leading to socially supportive and harmonious interpersonal relationships. Using the trust game, we previously reported sex differences in behaviour and alterations of trust behaviour, but our previous study was limited by lack of assessing biomarkers^16^. To our knowledge, this is the first report to show that interpersonal behaviour is significantly related with blood biomarkers, supplementing our previous study^16^.

Sex differences were consistently observed in the present cross-sectional study. Our study indicates that blood substances are likely to change behaviour (i.e., Cooperative) in males, and emotions (i.e., Trust) in females. There are gender differences in how people seek social support. Men tend to seek instrumental and tangible support, whereas women often seek emotional support^20,21^. Sex differences in the way people seek social support are possibly associated with psychosocial features of avoidant personality traits, leading to sex differences in our study. It would be effective to seek gender segregated treatment approaches for avoidant personality. For example, exercise or diet interventions, which affect UA and HDL-C levels, may be effective for men with avoidant personality traits. In contrast, women with avoidant personality traits may need psychotherapeutic approaches focusing on personality pathologies and trust. Differences between sexes are also possibly caused by multiple factors such as sex hormones and estrous cycles which influence mental functions including mood and emotion^22^, lifestyle differences, and cultures which accept sex differences in thought, cognition, behaviour, intrapersonal masculinity and femininity.

Oxidative stress and inflammation have recently been highlighted to understand not only pathological mechanisms of various psychiatric disorders but also socio-culture-based human behavior^22,23^. In Study 1, candidate blood biomarkers of avoidant personality traits were identified as follows: HDL-C and UA in men, and FDP and hsCRP in women. Interestingly, both HDL-C and UA are known as antioxidants. HDL-C has antioxidant and also anti-inflammatory functions^24^, and UA is a representative endogenous antioxidant widely erasing reactive oxygen species^25^. Oxidative stress decreases serotonin levels in the brain^26^ and has been highlighted as a candidate in the etiology of psychiatric diseases including depression^27^. For example, patients with major depressive and anxiety disorders have lower plasma UA levels^28^. Considering the association between dysregulation of the serotonergic system and avoidant personality disorder^29^, the decrease of antioxidants may induce social anxiety and avoidance behaviour via oxidative stress in men. Furthermore, previous studies have reported that both HDL-C and UA were associated with psychological and behavioural features. Low HDL-C was associated with specific personality traits characterized by self-centeredness, emotionality, and erratic behaviour^30^. Men with high serum UA levels are likely to have a hyperthymic temperament^31^. Economic generosity of males with higher HDL-C or UA found in the present study was possibly a result of altruistic traits and hyperthymic temperament. Both FDP and hsCRP are associated with inflammation. FDP has pro-inflammatory effects^32^, and hsCRP is widely used in clinical settings as a non-specific marker of acute inflammation. Inflammation has been recently gathering attention in the research field of various psychiatric problems including anxiety and depression^15,33^. The possible link between inflammation and avoidant personality traits is obesity. Obesity is positively correlated with inflammation^33^ and also avoidant personality disorder^34^. These findings suggest that avoidant personality traits may be due to inflammation, which influences emotions in women. The trust game conducted in the present study showed that women possibly judged whether somebody is trustworthy or not under the direct or indirect (i.e., through avoidant personality traits) influence of inflammation, proposing that inflammation affects the social decision-making system. We have previously reported that minocycline, an antibiotic drug that has an anti-inflammatory action via suppressing microglial activation, affected behaviour in the trust game^35^, suggesting the possibility that unconsciousness-based actions are modulated by brain inflammation^15^. These minocycline trials and the findings in the present study indicate that anti-inflammatory therapy is possibly important for treatment and prevention of avoidant personality-related problems including social withdrawal and its severe form, hikikomori. Further investigations on the association between inflammation and avoidant personality-related problems are required.

In Study 2, individuals with hikikomori had higher avoidant personality scores in both sexes. This trend suggests symptomatic similarities between hikikomori and avoidant personality disorder, and is congruent with the previous report that the most common comorbidity of hikikomori was avoidant personality disorder^11^. Our proposed blood biomarkers associated with hikikomori are UA in males and HDL-C in females. As discussed above, both UA and HDL-C have an antioxidative activity in common, suggesting that the phenomenon of hikikomori is possible associated with oxidative stress rather than inflammation in both genders contrary to expectations. The reason remains unclear, but we prospect that active social interaction may induce inflammation, and withdrawal from social interaction may ameliorate inflammation, causing individuals with hikikomori to have little inflammation.

The present study has several limitations. First, our study is based on relatively small samples recruited only in the same region, consequently resulting in selection bias. However, no reports exist comparing individuals with hikikomori and healthy controls, and analyzing multifaceted aspects of avoidant personality traits including behavioural features of more than 100 non-clinical persons. Therefore, we believe in the importance of our study. Second, we cannot exclude the possibility that some participants in Study 1 had psychiatric disorders, because structured diagnostic interviews for psychiatric disorders were not conducted for each healthy volunteer. In the present study, the possibility cannot be denied that some individuals with hikikomori have comorbidity with other psychiatric conditions such as depression and/or neurodevelopmental disorders. In future studies, structured diagnostic interviews such as the Mini-International Neuropsychiatric Interview (MINI)^36^ and the Autism Diagnostic Observation Schedule, Second Edition (ADOS-2)^37–39^ should be conducted in individuals with hikikomori, and comparison studies are warranted to clarify the similarity and differences of blood biomarkers between hikikomori and other psychiatric disorders. Third, we did not measure direct biomarkers regarding oxidative stress such as the total oxidant status and the total antioxidant status. Evaluating these direct oxidative stress-evaluating biomarkers might provide further strong evidence. Fourth, although individuals with hikikomori had lower serum levels of UA and HDL-C in the present study, their levels were all within normal limits. Recently, multidimensional evaluation systems using large data samples have been regarded to be important in developing useful biomarkers for diagnosis of psychiatric disorders^40,41^. Thus, the development of an evaluation method combining blood biomarkers with other biomarkers including brain imaging and behavioural characteristics is strongly needed for diagnosis and/or prediction of hikikomori. Fifth, some individuals with hikikomori in the present study took psychiatric medications, which may have influenced the present outcomes. Further investigation should be conducted considering the influence of medication in clinical samples. Sixth, this is a cross-sectional study not allowing any causal relationships between avoidant personality traits, blood biomarkers, and behavioural and psychological features. Therefore, we compensated this limitation by using SEMs. Finally, there might be some confounding factors such as tobacco use and eating/fitness habits. Hikikomori is suggested to be a phenomenon caused due to bio-psycho-social, cultural, or environmental etiologies^6^, all of which could be potential confounding factors. Hence, further studies are needed to validate our findings and elucidate the role of biomarkers in avoidant personality and hikikomori.

To conclude, we herein revealed the following aspects: inflammatory and antioxidant markers of HDL-C, FDP, UA and hsCRP were correlated with avoidant personality traits, cooperative behaviour and/or trust, and psychological features of isolation by gender. Individuals with hikikomori had higher avoidant personality traits in both genders, lower UA in men, and lower HDL-C in women. Based on the present findings, we suggest a novel hypothesis: avoidant personality may be formed by interactive relationships between oxidative stress and inflammation, and affects behavioural and psychological features, leading to the phenomenon of hikikomori. Therefore, we believe that the present study will shed new light on clarifying the underlying biological basis of hikikomori.

## Methods

The present study was carried out in accordance with the latest version of the Declaration of Helsinki and approved by the ethics committee of Kyushu University. All participants signed written informed consent to participate after a complete instruction of the study.

### Study 1

#### Participants

A university campus was selected as a place to advertise our study for recruiting young non-clinical volunteers. We used posters and flyers on the Kyushu University campus targeting students and staff as healthy volunteers. A total of 101 volunteers were recruited, and most of them were university students. After obtaining informed consent, their socio-demographic information was collected. A medical doctor carefully asked all participants regarding their physical condition. Exclusion criteria included age < 17, not proficient in Japanese, a self-reported history of psychiatric disorders including schizophrenia, traumatic brain injury, or severe heart, liver, or kidney disease.

#### Procedure

All participants completed a series of self-rated questionnaires, played a trust game^16,42^, and provided a non-fasting venous blood sample. Measured blood biomarkers were as follows: serum HDL-C, LDL-C, serum total bilirubin, UA, hsCRP, and plasma FDP. These biomarkers were adopted because all items could be easily measured in normal clinical settings and have been gathering attention in the research field of various psychiatric diseases including schizophrenia^43–47^. Some biomarkers were selected for future comparison with the large cohorts, the Midlife in the United States and the Midlife in Japan^48^. Although tested samples were non-fasting venous blood, effects of meal on serum LDL-C and HDL-C were thought to be negligible^49^.

#### Self-rated Questionnaires

All participants completed the Structured Clinical Interview for the Diagnostic and Statistical Manual of Mental Disorders, Fourth Edition (DSM–IV) Personality Disorders Personality Questionnaire (SCID-II/PQ), a 119-item questionnaire version of the SCID-II, on which the items are answered using a “yes (=1 point)” and “no (=0 point)” response format^50^. The items relevant to each personality disorder were summed for a total score defined as an index of each personality trait. We evaluated avoidant personality scores in participants with SCID-II/PQ (Max. 7 points for the personality trait). Moreover, considering common psychological and behavioural features of avoidant personality disorder and hikikomori, we administered the following questionnaires: PSS, assessing one’s preference for solitude^51^; R-UCLA, assessing one’s degree of loneliness^52,53^; YYS, measuring respondents’ estimation of others’ trustworthiness^54^; IAT, assessing the degree to which Internet use affects daily routine, social life, productivity, sleeping pattern and feelings^55^; LSNS-6, gauging social isolation by assessing the number of people in one’s social network with whom one has social contact and social support^56,57^; MSPSS, measuring the perceived adequacy of support from family, friends and significant others^58^; and MINI-SPIN, having good efficiency in distinguishing individuals with generalized social anxiety in a health care setting^59,60^.

#### PC-based Trust Game

We evaluated trusting behaviour of participants towards others by a trust game, conducted on PCs based on our previous study^16^. In this two-player game, each player initially receives an explanation of the game rules and is given 1300 yen (JPY) [about 12 USD]. The first player then decides how much of the 1300 JPY to give to the second player (partner) and rates the partner’s attractiveness and trustworthiness based on their photographs presented on a computer screen (both of the score ranges: 0–9). The amount of money given to the partner by the first player (i.e., Monetary Scores) is tripled. The second player then decides whether to split the money equally with the first player or take away the entire amount of money. In this PC experiment, all the participants were actually assigned to be the first player, and the second players were virtual players on a computer screen. The participants had no information about their partners except for the facial photographs including the head and shoulders with a neutral facial expression. Photos of second players were selected from professional fashion models (i.e., “high attractive partners”) or lay individuals (i.e., “ordinary attractive partners”). We randomly selected 40 pictures (10 each of professional male fashion models, professional female fashion models, lay males, and lay females) for the trust game. The first player was not aware of the partner’s decision. The first player’s decision as to how much money to give to the second player is thought to reflect their level of trust in the second player. After the experiments, each participant was actually paid the amount of money corresponding to the result of a randomly selected game from all games as a reward.

#### Statistics

The path analysis and the other analyses were performed by SPSS AMOS version 24.0 and SPSS version 22 (SPSS, Inc., Chicago, IL, USA), respectively. All analyses were stratified by participant gender. Differences in the demographic data between both genders were analyzed using chi-squared test, Welch’s t test, or Mann-Whitney U test. First, we assessed causal relationships between avoidant personality score, blood biomarkers, psychometrics, and behavioural characteristics by using SEM. The latent variables consisted of (i) “Isolation”: PSS, R-UCLA, IAT, LSNS-6, and MSPSS; (ii) “Interpersonal Psychology”: YYS and MINI-SPIN; (iii) “Cooperativeness”: Monetary Scores; (iv) “Trust”: Trustworthiness; (v) “Affection”: Attractiveness. Various models were generated and assessed using the following fitting criterion: χ2, GFI, CFI, TLI, and RMSEA. χ2 is so-called ‘a badness of fit’ (we obtain a good fit model if *p* value is greater than 0.05). GFI greater than 0.90, CFI greater than 0.95, TLI greater than 0.95, and RMSEA less than 0.08 indicate an acceptable fit. GFI greater than 0.95, CFI greater than 0.97, TLI greater than 0.97, and RMSEA less than 0.05 indicate a good fit^61^. We compared the models to obtain the best-fit model using the parameter of Akaike’s Information Criterion. Second, multiple regression analyses were performed with stepwise method for efficient biomarkers detection. All blood biomarkers were the independent variables, and avoidant personality score was the dependent variable. To avoid the issue of multicollinearity, we chose the stepwise option and confirmed that the scores of condition indices were below fifteen for each final model. Finally, to calculate Pearson’s correlation coefficients (r) or Spearman’s correlation coefficients (ρ), correlation analyses were performed between avoidant personality score, blood biomarkers, psychometrics, and behavioural characteristics. We used a two-tailed significance level of *p* = 0.05 and a trend level of *p* = 0.1 throughout the study.

### Study 2

#### Participant

This case-control study was conducted in Fukuoka, a major metropolitan area in southern Japan. Study recruitment occurred in six psychiatric hospitals and clinics affiliated with the Department of Neuropsychiatry of Kyushu University, and two community mental health centers. We included all individuals from the eight clinical sites who met criteria for currently having hikikomori based on a structured diagnostic interview designed for this project. Exclusion criteria were age <15 or >50, a self-reported history of schizophrenia, traumatic brain injury, or severe heart, liver, or kidney disease. For healthy control group recruitment, we used posters and flyers on the Kyushu University campus. Healthy controls were who: (i) did not have a history of current hikikomori or past physical isolation; and (ii) did not meet criteria for any current psychiatric disorder as per the Structured Clinical Interview for the DSM–IV Axis I Disorders. Exclusion criteria were age <15 or >50, a self-reported history of any psychiatric disorders, traumatic brain injury, or severe heart, liver, or kidney disease.

#### Procedure

All participants completed a series of self-rated questionnaires same as conducted in Study 1, underwent a structured interview for hikikomori with a study psychiatrist or psychologist, and provided a non-fasting venous blood sample. The structured interview had been developed for hikikomori in our prior theoretical work and empirical research^11,62^. Criteria for hikikomori were as follows: (i) spending most of the time at one’s home or residence at least for six months; (ii) significant distress or impairment associated with the social withdrawal; and (iii) a physical or medical etiology is not the primary reason for the social withdrawal. Measured blood biomarkers were same as in Study 1 for the same reasons.

#### Statistics

All analyses were performed by SPSS version 22 (SPSS, Inc., Chicago, IL, USA) and stratified by participant gender. Differences in age between two groups were analyzed using Welch’s t test or Mann-Whitney U test. First, we assessed normality of data using Shapiro-Wilk test, and then performed Mann-Whitney U test or Student’s/Welch’s t test to compare avoidant personality score and blood biomarkers between the two groups. Second, to assess the classifying efficiency of each blood biomarker, we performed canonical discriminant analysis using hikikomori diagnosis as the dependent variable, and candidate biomarkers based on the comparisons between two groups as the predictor variables. Cross-validation procedure by leave-one out classification was performed in this discriminant analysis. Moreover, ROC curves were constructed to assess the potential of candidate biomarkers. Finally, to calculate Pearson’s correlation coefficients (r) or Spearman’s correlation coefficients (ρ), correlation analyses were performed between avoidant personality score, blood biomarkers, and psychometrics. We used a two-tailed significance level of *p* = 0.05 and a trend level of *p* = 0.1 throughout the study.

## Electronic supplementary material

Supplementary information

Supplementary Table 1

Supplementary Table 2

Supplementary Table 3

Supplementary Table 4

Supplementary Table 5

Supplementary Table 6

Supplementary Table 7
