# Supplementary material for: Blood biomarkers of Hikikomori, a severe social withdrawal syndrome
Source: Sci Rep. 2018 Feb 13;8:2884. doi: 10.1038/s41598-018-21260-w (PMC5811600; doi:10.1038/s41598-018-21260-w)
Supplement: Supplementary file 1 — Supplementary information [file 41598_2018_21260_MOESM1_ESM.pdf]

Title: Blood biomarkers of Hikikomori, a severe social withdrawal syndrome

Authors:

Kohei Hayakawa, M.D.[1], Takahiro A. Kato, M.D., Ph.D.[1*], Motoki Watabe, Ph.D.[2#], Alan R. Teo, M.D., M.S. [3&4#], Hideki Horikawa, M.D., Ph.D.[1], Nobuki Kuwano, M.D.[1], Norihiro Shimokawa, M.D.[1], Mina Sato-Kasai, M.D.[1], Hiroaki Kubo, M.A.[1], Masahiro Ohgidani, Ph.D.[1], Noriaki Sagata, Ph.D.[1], Hiroyuki Toda, M.D., Ph.D.[5], Masaru Tateno, M.D., Ph.D.[6], Naotaka Shinfuku, M.D., Ph.D.[7], Junji Kishimoto, Ph.D.[8], Shigenobu Kanba, M.D., Ph.D.[1]

Affiliations:

[1] Department of Neuropsychiatry, Graduate School of Medical Sciences, Kyushu University, Fukuoka, Japan

[2] School of Business, Monash University, Jalan Lagoon Selatan, Bandar Sunway, Selangor Darul Ehsan, Malaysia

[3] VA Portland Health Care System, Portland, Oregon, United States of America

[4] Department of Psychiatry, Oregon Health & Science University, Portland, Oregon, United States of America

[5] Department of Psychiatry, National Defense Medical College, Tokorozawa, Saitama, Japan

[6] Department of Neuropsychiatry, Sapporo Medical University, School of Medicine, Sapporo, Hokkaido, Japan

[7] International Center for Medical Research, Kobe University, Kobe, Japan

[8] Department of Research and Development of Next Generation Medicine, Faculty of

Medical Sciences, Kyushu University, Fukuoka, Japan

(#. These authors are equally contributed.)

***Correspondence to:**

Takahiro A. Kato, M.D., PhD.

Associate Professor

Department of Neuropsychiatry, Graduate School of Medical Sciences, Kyushu University

Address: Maidashi 3-1-1, Higashi-ku, Fukuoka 812-8582, Japan

Phone: +81-92-642-5627, Fax: +81-92-642-5644

E-mail address: takahiro@npsych.med.kyushu-u.ac.jp

Supplementary Table 1

Avoidant personality score and biomarkers/psychometrics in non-hikikomori volunteers.

Supplementary Table 2

Avoidant personality score and trust game in non-hikikomori volunteers.

Supplementary Table 3

Blood biomarkers and psychometrics/trust game in male non-hikikomori volunteers.

Supplementary Table 4

Blood biomarkers and psychometrics/trust game in female non-hikikomori volunteers.

Supplementary Table 5

Discriminant analysis and ROC curves using candidate biomarkers between individuals with hikikomori and age-matched controls.

Supplementary Table 6

Blood biomarkers and avoidant personality score/psychometrics in male individuals with hikikomori.

Supplementary Table 7

Blood biomarkers and avoidant personality score/psychometrics in female individuals with hikikomori.
